# Rash and Fever: Identifying Animal or Plant Origins

**DOI:** 10.7759/cureus.77654

**Published:** 2025-01-19

**Authors:** Sabina Azevedo, Sara Pereira, Rita Vilar da Mota, Rafael Lopes Freitas, Liliana Alves Costa

**Affiliations:** 1 Internal Medicine, Unidade Local de Saúde Alto Minho, Ponte de Lima, PRT; 2 Internal Medicine, Unidade Local de Saúde Alto Minho, Viana do Castelo, PRT

**Keywords:** dermatitis, dermatology, furocoumarins, internal medicine, photoreaction, photosensitivity disorders, phytochemicals, phytophotodermatitis, solar radiation

## Abstract

Phytophotodermatitis is a dermatological condition resulting from exposure of the skin to furanocoumarins and subsequent exposure to ultraviolet radiation. It has been described mainly after direct contact with plants but also with their extracts, particularly limes, lemons, figs, celery, or parsley. The clinical presentation is variable and may range from asymptomatic to erythematous rash, which may progress into vesicles or bullae. The main differential diagnoses are contact dermatitis, allergic dermatitis, and burns. The diagnosis is based on clinical history, clinical findings, and physical examination. In such cases, accurate diagnosis is necessary to avoid unnecessary treatment. We present the case of a 55-year-old patient with recurrent episodes of pruritus who presented with a new rash and fever. After a review of the aetiology and clinical history, supported by a skin biopsy, a diagnosis of phytophotodermatitis with secondary bacterial infection was made. After treatment with antibiotics and corticosteroids, the symptoms disappeared, and a non-pharmacological approach was recommended. The aim of this article is, therefore, to emphasize the significance of this disorder, to raise awareness of it, and to emphasize the importance, in an increasingly technological age, of a detailed clinical history and physical examination as the main tool for a correct diagnosis.

## Introduction

Phytophotodermatitis is a dermatological condition characterized by a non-immune phototoxic reaction, which results from exposure to furanocoumarins, followed by exposure to ultraviolet A (UVA) solar radiation [1-6]. Furanocoumarins are phototoxic substances composed of psoralen isomers, which remain inactive until exposed to sunlight. They are mainly found in plants from the *Rutaceae, Apiaceae, Moraceae,* and *Fabaceae* families [1], such as limes, lemons, figs, celery, and parsley [3,5,7]. After the exposure, a photosensitivity reaction is induced with the release of reactive oxygen species, damage to DNA and RNA chains in cells, and consequent damage to the cell membrane [3,6,7]. This intense inflammatory reaction culminates in the characteristic symptoms of phytophotodermatitis.

Typically, skin lesions appear eight to 24 hours after exposure and are widely variable [6]. Initially, the lesions are often asymptomatic but may evolve in later stages to erythematous rash and, over time, become hyperpigmented areas [4]. The most common presentation is in the form of erythematous macules and streaks on the exposed areas, accompanied by a burning sensation, and may progress to an oedematous phase with vesicles and bullae [3,6]. Pruritus may occur but is not a major manifestation, unlike allergic phytodermatitis. The diagnosis of phytophotodermatitis is clinical and based on a thorough history of exposure to plants and sunlight [2]. This condition is often confused with a wide range of dermatological conditions, particularly sunburn, hypersensitivity reactions, or contact dermatitis [4].

The authors present the case of a 55-year-old man with several episodes of skin rash over the years without any previous dermatologic diagnosis.

## Case presentation

**Figure 1:**
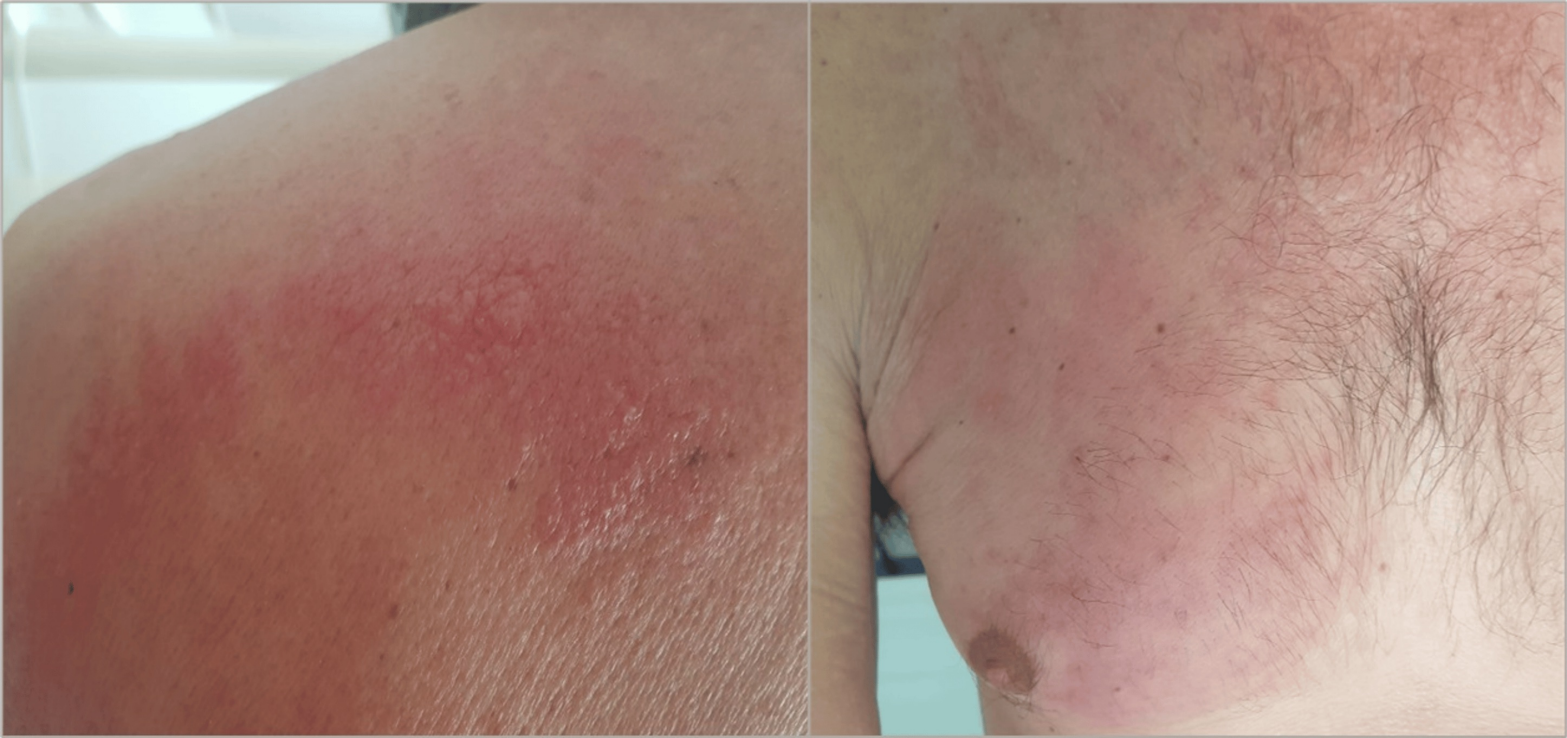
Erythematous and maculopapular lesions located on the posterior thorax, along with erythematous lesions observed on the anterior thorax.

**Figure 2:**
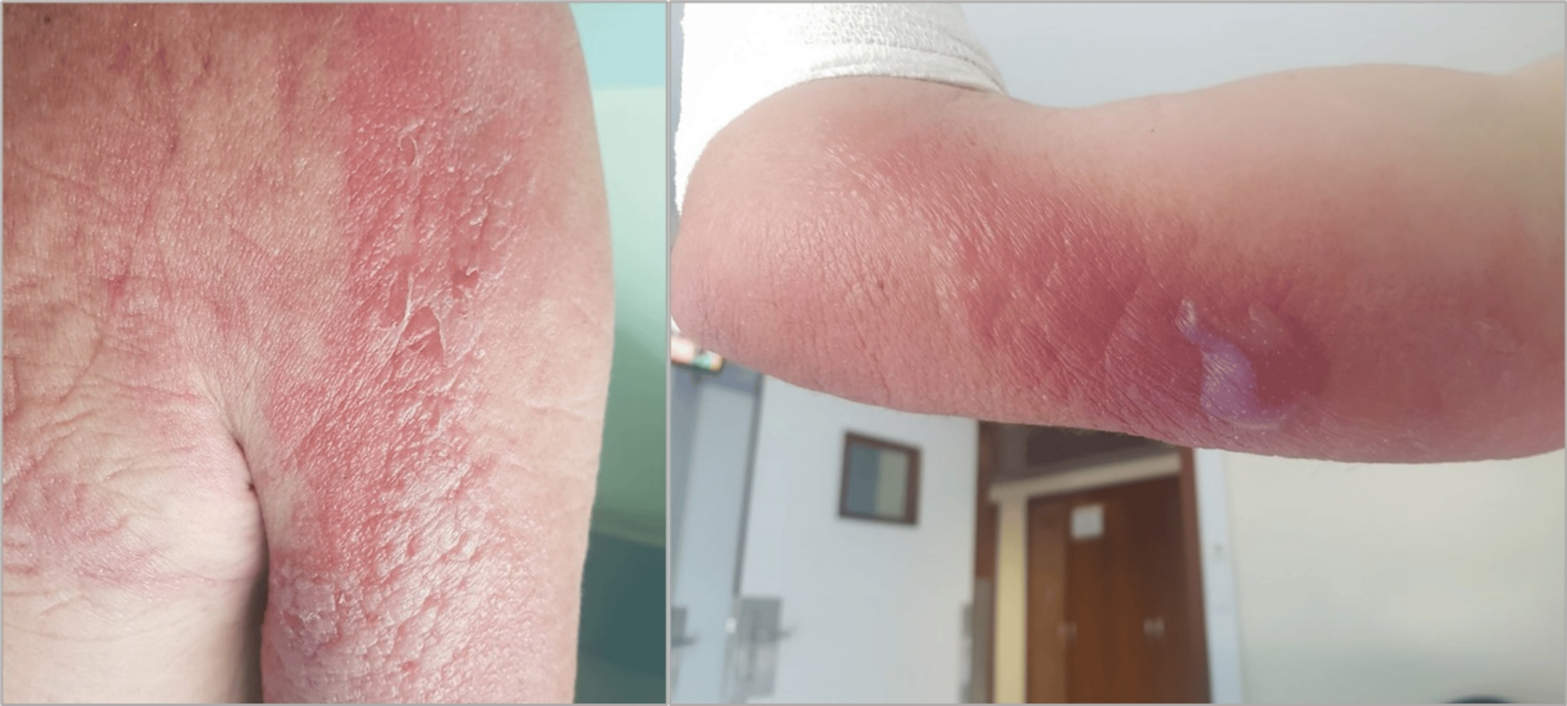
Erythematous-edematous lesions featuring vesicles and bullae on the upper extremities.

A 55-year-old man who works as a construction worker and farms in his spare time presented to the emergency department complaining of fever and pruritic rash for about one week. He had reported several similar episodes over several years without ever seeking medical attention. Upon initial assessment, the patient presented with fever and erythematous plaques with papular and vesicular eruptions scattered over the chest (Figure 1) and upper limbs (Figure 2). Laboratory tests showed elevated inflammatory markers, including a C-reactive protein level of 26 mg/dL and an erythrocyte sedimentation rate of 48 mm/h, with no other abnormalities noted (autoimmune screening was unavailable in the emergency department). A soft tissue ultrasound was made, and subcutaneous oedema was demonstrated without signs of thrombosis or abscess. He was admitted under suspicion of either zoonosis or erythema with bacterial overinfection and was started on antibiotic therapy with ceftriaxone. 

After a detailed clinical history, the patient reported that the day before the rash appeared, he had been doing agricultural work and clearing a forest, in contact with several plants and flowers that he couldn't specify, in the sun and without a shirt. Contact with various foods, hygiene products, or cosmetics was ruled out, as was the use of new foods or medicines. It was also noted that the previous episodes had always occurred on the trunk, in spring or summer, and after working in the fields or woods. The patient was unable to specify which plants he had come into contact with, only mentioning that there were several different species in the field. With the details of the clinical elements and the assessment by the dermatology department, the suspicion of the possibility of phytophotodermatitis increased, and topical corticotherapy was started after the infection had resolved.

During hospitalization, additional investigations were performed to identify the etiology. These included blood cultures, viral serologies for hepatitis, human immunodeficiency virus (HIV), herpes simplex virus types 1 and 2 (HSV-1, HSV-2), cytomegalovirus (CMV), and Epstein-Barr virus (EBV). Testing for zoonotic agents was conducted using serologies and polymerase chain reaction (PCR) for *Leptospira, Rickettsia, Toxoplasma gondii, and Brucella*. Autoimmune screening was also performed, including tests for antinuclear antibodies (ANA), rheumatoid factor (RF), and cytoplasmic antineutrophil cytoplasmic antibody (c-ANCA). All exams were negative for acute infection and for the suspicion of autoimmune disease. A thoracoabdominal-pelvic computed tomography scan was also performed with no acute findings. Finally, a skin biopsy of the lesions showed dermatitis with exuberant edema, superficial polymorphic inflammatory infiltrate without any eosinophils, and parakeratosis of the epidermis. These alterations, although characteristic of several dermatoses in the context of the presented case, allowed for the exclusion of other differential diagnoses, such as Wells syndrome or allergic dermatitis, and supported the final diagnosis of phytophotodermatitis.

After initiating topical corticosteroid treatment, the patient's clinical evolution improved, with apyrexia decreasing, inflammatory parameters decreasing, and the rash resolving. The patient was discharged with advice to avoid activities involving sun exposure and to use adequate sun protection when necessary. On reassessment after one month, he was apyretic, with areas of hyperpigmentation where he had previously had a rash and no analytical changes.

## Discussion

Cutaneous exposure to plants containing furocoumarins primarily occurs during outdoor activities, such as fig harvesting, daisy preparation, and contact with plants like *Heracleum spp. or Citrus spp.* Consequently, forestry workers, farmers, gardeners, and campers are frequently prone to developing skin rashes after contact with plant material [6]. Examples of such exposures include lake diving [2], fig harvesting [4], or recreational activities such as rock climbing [8]. However, plants are also found in indoor environments, and their extracts and derivatives are widely used in processed foods and fragrances [6], meaning that any individual who comes into contact with these products may also be affected, as evidenced by a reported case following the preparation of daisies [3]. Thus, the classic presentation of phytophotodermatitis often occurs during the warmer months or after holidays when there is a higher concentration of furocoumarins and increased UVA radiation [2,4].

The severity of symptoms depends on the characteristics of the exposure, particularly the amount of phototoxic substances and the duration of UVA exposure [2]. Early recognition of the condition with suggestive cutaneous changes is essential to prevent progression to chronic and difficult-to-resolve stages, as acute cutaneous manifestations that do not resolve spontaneously may develop into areas of hyperpigmentation lasting weeks to months [3,6,7]. These changes are the result of delayed post-inflammatory reactions leading to melanogenesis stimulation induced by psoralen, a naturally occurring compound found in furocoumarin [3]. Despite this, phytophotodermatitis is typically a self-limiting condition with no long-term sequelae [4,7].

There are a lot of differential diagnoses, such as irritant contact dermatitis, photosensitivity disorders (pellagra, solar urticaria, and porphyria), infections (tinea and herpes simplex virus), drug-induced allergic reactions, erythema multiforme, or vesiculobullous disorders [1]. One of the main differential diagnoses is photoallergic dermatitis [2-4], a delayed hypersensitivity reaction mediated by immune mechanisms [4], as opposed to phytophotodermatitis, which is caused by direct cellular damage by phototoxic substances following UVA exposure [1,6]. Although the cutaneous manifestations are similar, certain distinguishing features are important, including the presence of intense pruritus, which is more typical of an allergic reaction. Phytophotodermatitis lesions occur only in areas exposed to sunlight, and the resolution of these lesions is often accompanied by areas of hyperpigmentation, which is not seen in allergic reactions [2]. Although primarily a clinical diagnosis, certain tests may be helpful, such as photopatch testing [3,7] or skin biopsies [1].

The approach focuses on symptomatic treatment and prevention of future events [4]. Supportive treatment includes cold compresses, analgesia, and local antiseptics to prevent secondary bacterial infections [1-3]. Moderate to severe cases may require topical or oral corticosteroids and antibiotics if secondary bacterial infection is suspected [1-9]. More severe cases may require referral to dermatology [2-4,7]. Non-pharmacological advice to prevent recurrence is crucial, including the use of UVA protection, physical barriers, and sunscreens, as well as avoiding contact with plants and hand hygiene [4,8]. Despite being a relatively common condition with simple treatment, it remains under-recognized, and awareness is important for the prevention and safety of professionals such as gardeners or forest workers [9].

## Conclusions

The case presented highlights the importance of taking a detailed history when evaluating patients with cutaneous manifestations. Given the wide spectrum of these manifestations, both in dermatological conditions and in other systemic diseases, clinical suspicion of phytodermatitis is crucial, as it is highly likely to be confused with other conditions such as contact dermatitis, phytodermatitis or photoallergic dermatitis, burns or cellulitis. In order to establish the diagnosis, it is essential to take a thorough history with a detailed questioning of the patient's exposure to plants or their derivatives, as well as the recent epidemiological context of the patient, especially during the warmer months or after holiday travel.

An accurate diagnosis is crucial for proper treatment and the implementation of preventive measures. In fact, asymptomatic or mild forms of the disease do not require systemic treatments, such as antibiotic therapy or oral corticosteroids, and their use should be reserved for severe presentations and complications. It is also important to emphasize the need for non-pharmacological advice, such as wearing protective clothing or promptly washing exposed skin after suspected plant contact, to prevent this condition properly. Thus, despite its association with common plants, public awareness of this condition remains low. Therefore, this article aims to remind the medical community, especially non-dermatologists, of the existence of this condition and the importance of maintaining a detailed history, clinical assessment, and physical examination as the primary diagnostic tools in an era increasingly dependent on technology and diagnostic tests.
